# Cellular interactions in the pituitary stem cell niche

**DOI:** 10.1007/s00018-022-04612-8

**Published:** 2022-12-01

**Authors:** Thea L. Willis, Emily J. Lodge, Cynthia L. Andoniadou, Val Yianni

**Affiliations:** 1 Centre for Craniofacial and Regenerative Biology, Faculty of Dentistry, Oral and Craniofacial Sciences, King’s College London, London, UK; 2 Department of Medicine III, University Hospital Carl Gustav Carus, Technische Universität Dresden, Dresden, Germany

**Keywords:** Stem cell, Pituitary, SOX2, Niche, Paracrine, Single cell RNAseq

## Abstract

Stem cells in the anterior pituitary gland can give rise to all resident endocrine cells and are integral components for the appropriate development and subsequent maintenance of the organ. Located in discreet niches within the gland, stem cells are involved in bi-directional signalling with their surrounding neighbours, interactions which underpin pituitary gland homeostasis and response to organ challenge or physiological demand. In this review we highlight core signalling pathways that steer pituitary progenitors towards specific endocrine fate decisions throughout development. We further elaborate on those which are conserved in the stem cell niche postnatally, including WNT, YAP/TAZ and Notch signalling. Furthermore, we have collated a directory of single cell RNA sequencing studies carried out on pituitaries across multiple organisms, which have the potential to provide a vast database to study stem cell niche components in an unbiased manner. Reviewing published data, we highlight that stem cells are one of the main signalling hubs within the anterior pituitary. In future, coupling single cell sequencing approaches with genetic manipulation tools in vivo, will enable elucidation of how previously understudied signalling pathways function within the anterior pituitary stem cell niche.

## Introduction

An intense area of research in regenerative biology is the environment supporting stem cell maintenance, self-renewal and differentiation i.e. the stem cell niche. In epithelial structures such as the pituitary gland [1, 2], intestine [3], and pancreas [4], every individual niche is composed of organ-specific stem cells, progenitors, differentiated progeny, and other supporting cell types such as mesenchymal cells, endothelial cells, and immune cells, as well as extracellular matrix (ECM). Within this niche, there exists a complex interplay between short- and long-range signals that govern the behaviour of stem cells and their descendants [5]. These include secreted factors and cell-to-cell interactions, mediated by cell membrane proteins, or direct interactions with the ECM. All of the above, culminate in the activation of key transcription factors (TFs) that activate cell type-specific gene expression programs. In this review, we summarise current knowledge on pituitary stem cells (PSCs) and their niche, PSC cellular interactions and novel methods whereby we can interrogate PSC identity and function.

**Fig. 1:**
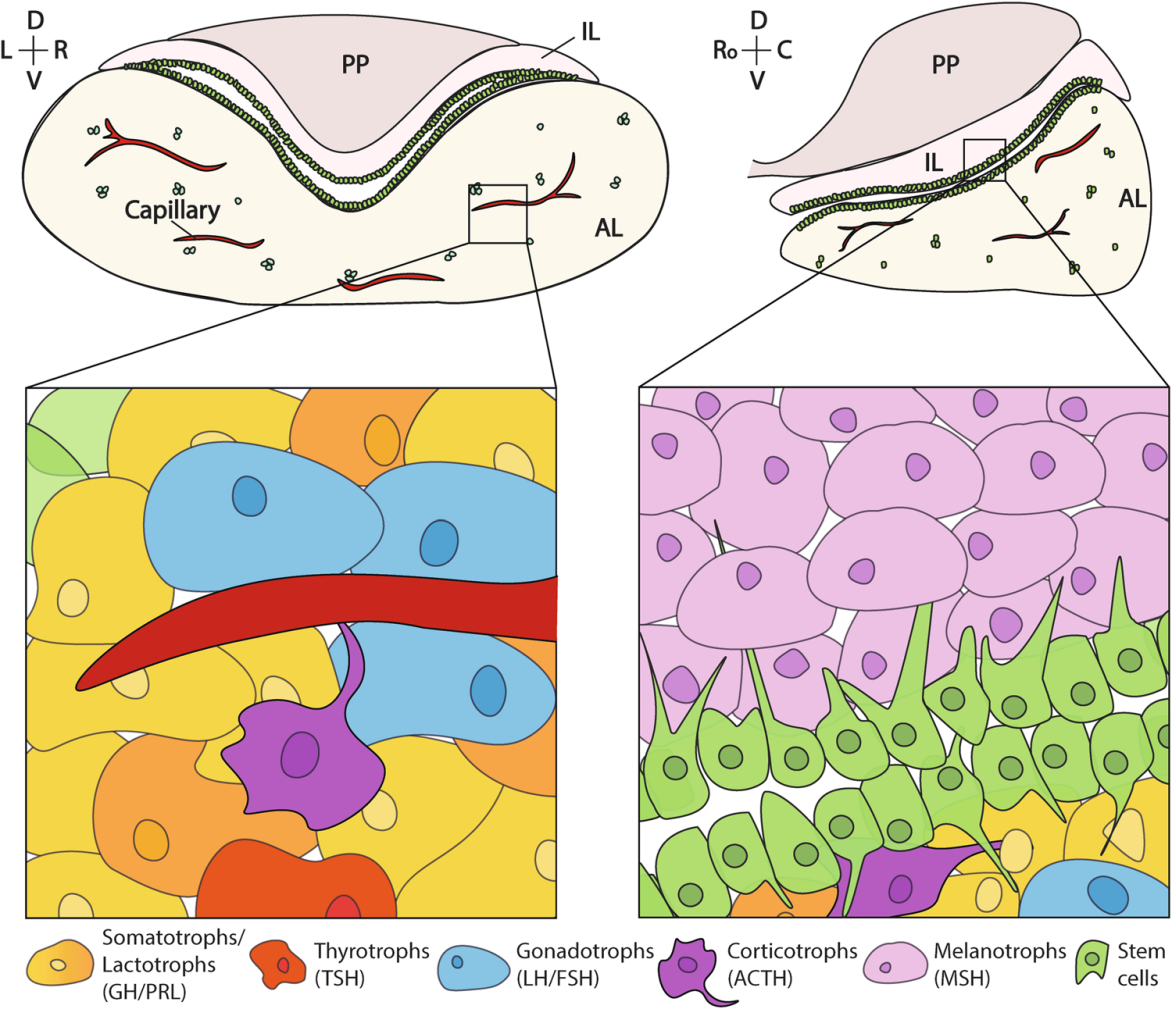
Schematic of the postnatal murine pituitary. The postnatal pituitary gland consists of the Posterior Pituitary (PP) and the Anterior Pituitary (AP), the latter of which houses the anterior lobe (AL) and intermediate lobe (IL). Hormone-producing cells, resident stem cells and non-hormonal support cells such as endothelia and connective tissue are present with the AP. Melanotrophs (secreting MSH) are present uniquely within the IL, whilst Lactotrophs (PRL), Somatotrophs (GH), Thyrotrophs (TSH), Gonadotrophs (LH/FSH) and Corticotrophs (ACTH) are present within the parenchyma of the AL. Stem cells reside within the marginal zone between the IL and the AL in the AP and as epithelial clusters within the parenchyma of the AL. Vasculature is present throughout the AP. For the axes, *D* dorsal, *V* ventral, *L* left, *R* right, *Ro* rostral, *C* caudal.

**Fig. 2:**
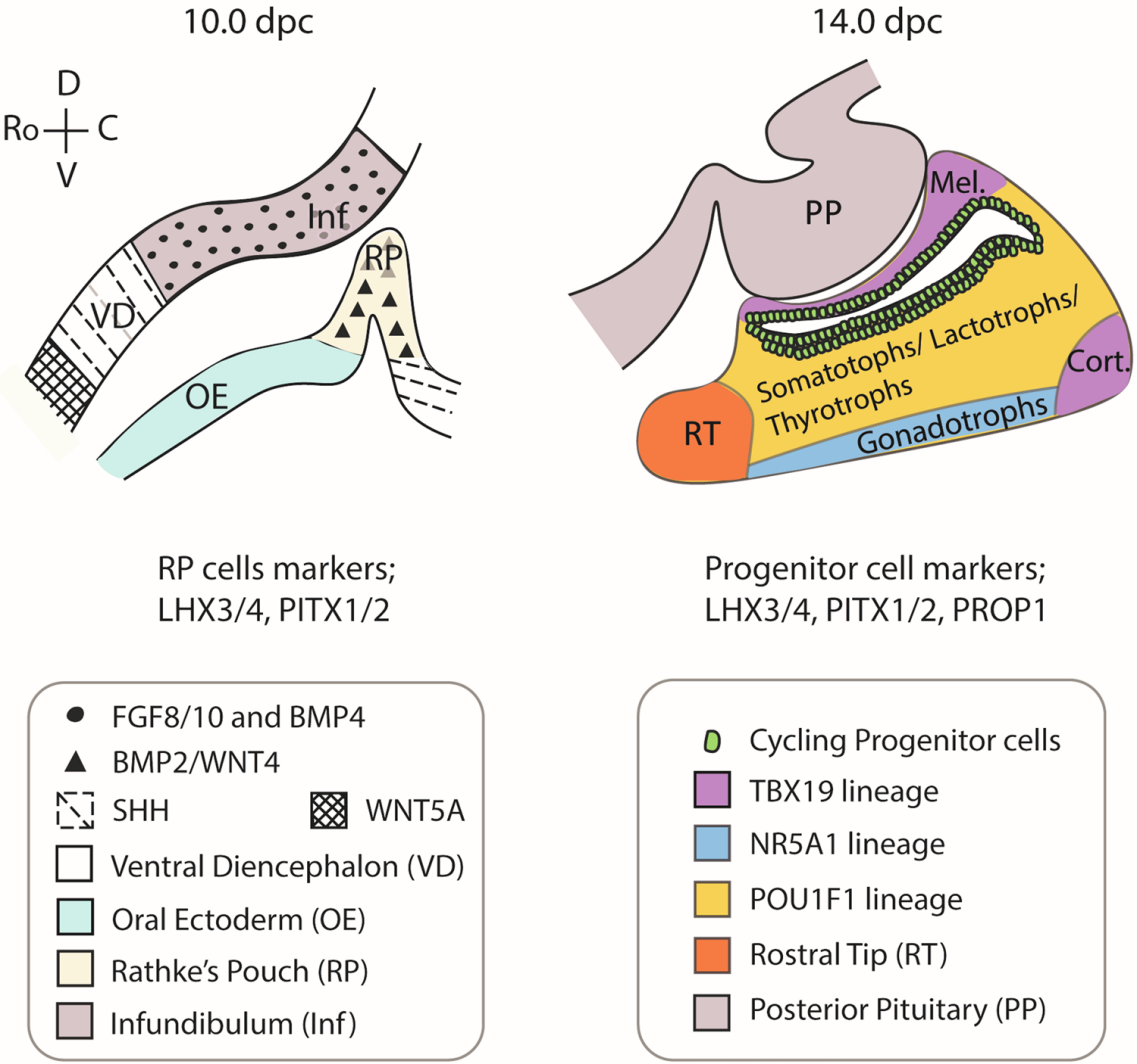
Signals and cell locations during mouse pituitary organogenesis. Thickening of the oral ectoderm (OE) at 8.5 dpc results the formation of Rathke’s Pouch (RP) by 9.0 dpc. This structure, orchestrated by signals from the neural ectoderm/ventral diencephalon (VD), goes on to become the developing pituitary gland. Signalling of the WNT, BMP, FGF and Sonic hedgehog (SHH) pathways is spatially restricted and is crucial for proper pituitary patterning. At 10.0 dpc all cells of RP are marked by LHX3/4 and PITX1/2. By 14.0 dpc it is mainly the progenitor cells, present in the more dorsal part of the pituitary that express these markers as well as PROP1. At this timepoint, hormone secreting POU1F1-independent thyrotrophs are present in the rostral tip (RT) and other lineage-restricted cells are beginning to appear. Italicised hormone-producing cell names indicate their future location within the developing gland. *Mel* Melanotrophs, are present within the intermediate lobe and *Cort* Corticotrophs are situated more ventrally. Compartments are shaded by lineage restriction outlined in the age-specific key. *PP* posterior pituitary, *D* dorsal, *V* ventral, *Ro* rostral, *C* caudal.

The pituitary gland has an indispensable role in fine-tuning multiple facets of physiology as it regulates growth, sexual maturity & reproduction, the stress response, and metabolism. The response to hormonal demand relies on integrating incoming signals from the hypothalamus. Therefore, the concerted development of the two tissues is critical. The mature pituitary gland consists of the posterior pituitary (PP, neurohypophysis) and the anterior pituitary (AP, adenohypophysis). The PP includes axon terminals originating from the hypothalamus via the pituitary stalk. The dual function of the PP is to store and secrete antidiuretic hormone and oxytocin, which are produced by these neurons, into the blood stream, alongside relaying stimulatory signals from the hypothalamus to the anterior pituitary, promoting hormone release [6]. The AP contains the majority of hormone-secreting endocrine cells (Fig. 1). These include prolactin (PRL)-secreting lactotrophs, growth hormone (GH)-secreting somatotrophs, thyroid-stimulating hormone (TSH)-secreting thyrotrophs, luteinising hormone (LH)- and follicle-stimulating hormone (FSH)-secreting gonadotrophs, and adrenocorticotrophic hormone (ACTH)-secreting corticotrophs. Additionally, in the mouse, melanotrophs reside in the intermediate lobe (IL) and secrete melanocyte-stimulating hormone (MSH) [7, 8], whilst all other committed cells reside within the anterior lobe (AL) of the AP (Fig. 1). All of these hormone-producing cell types derive from three distinct lineages of progenitors, distinguished by their determining transcription factors; the POU1F1 (previously PIT1) lineage (somatotrophs, lactotrophs and thyrotrophs), TBX19 (previously TPIT) lineage (corticotrophs and melanotrophs) and NR5A1 (previously SF1) lineage (gonadotrophs) (Figs. 1, 2).

In rodents, pituitary development is initiated when a thickening in the oral ectoderm, expressing transcription factors PITX1/2, invaginates and bulges towards the ventral diencephalon, derived from neural ectoderm [9–12]. Reciprocal signalling from both tissues is required for this process. This anatomical structure is referred to as the anterior pituitary anlage or primitive ‘Rathke’s pouch’ (RP), which consists of uncommitted progenitor cells that do not yet produce hormones [13]. A region of the ventral diencephalon also evaginates towards RP, forming the infundibulum or future posterior pituitary, acting as a major signalling centre during pituitary organogenesis [14–19] (Fig. 2). As development proceeds, RP further expands, closes on the ventral aspect and gradually detaches from the oral ectoderm to become the definitive RP, which will form the anterior pituitary. In the postnatal AP, in addition to the mature hormone-producing cells, resides a discreet population of long-lived pituitary stem cells (PSCs), which remains from the embryonic uncommitted progenitor pool that initially line the cleft and form the marginal zone epithelium (Fig. 2) [20, 21], as well as a number of non-endocrine cells such as endothelial, immune and mesenchymal cells. Human pituitary development closely mirrors pituitary development in rodents, in terms of the major tissue interactions and signals [22]. In this review we describe the major signalling events that pattern the embryonic pituitary gland and how perturbation of these impacts on development. Moreover, we focus on the signalling mechanisms that regulate the postnatal stem cell niche in the anterior pituitary, ensuring the dynamic turnover and adaptability of the organ to physiological demand. Lastly, we highlight recent advances in single cell technologies and how they are expanding our understanding of pituitary stem cell heterogeneity, and inter-cellular interactions between the different endocrine cell populations.

## Signals regulating pituitary gland development

The early phase of pituitary development is a cooperative interaction consisting of multiple molecular mechanisms directing patterning, proliferation and commitment of progenitor cells. In part, this is achieved by key signalling centres arising during development that set up signalling gradients [23] (see Fig. 2). These gradients direct spatially- and temporally-restricted gene expression patterns, which ultimately lead an apparently uniform layer of ectodermal progenitors, becoming organised into the mature pituitary cell types. Major signalling pathways including FGF, BMP, WNT, SHH, Retinoic acid (RA), Notch and YAP/TAZ have all been identified as integral for pituitary organogenesis. While here we focus on these pathways in development, it is key to note that they are conserved and maintain a crucial role in regulation of the postnatal anterior pituitary and its PSC compartment.

In mouse ontogeny, pituitary organogenesis initiates with a thickening of the epithelial, oral roof ectoderm at 8.5 days post coitum (dpc). Multiple members of the LIM homeodomain family of transcription factors have been shown to initiate expression in RP during the early invagination stage, including LHX3, LHX4 and ISL-1. *Lhx3*^*−/−*^ mutant mice display a failure in ectodermal cell proliferation at 9.5 dpc, and a loss of all pituitary cell types [24]. To compare, *Lhx4*^*−/−*^ mutants display a hypoplastic anterior lobe, but all five committed cell lineages are present, albeit in reduced numbers. *Lhx4*^*−/−*^ null mice have arrested pituitary development on a *Lhx3* heterozygous background, indicating a potentially redundant role between LHX3 and LHX4 [25]. *Isl-1* shows broad expression across RP epithelium but becomes restricted in expression to ventral cell populations by 10.5 dpc. In *Isl-1*^*−/−*^ mutants, the initial invagination of the pouch occurs normally but the absence of *Isl-1* leads to a block in cellular proliferation and RP growth arrest at 9.5 dpc [14]. As the oral ectoderm invaginates and begins formation of Rathke’s pouch (9.0 dpc), a portion of the ventral diencephalon (neural ectoderm) begins to evaginate ventrally forming the infundibulum. The two structures make direct contact, with the infundibulum becoming the first key organising centre that plays a role in patterning the dorsal portion of Rathke’s pouch (Fig. 2). The necessity for the neural ectoderm in Rathke’s pouch specification was solidified with studies using the *Nkx2-1* (*T/ebp*) homozygous null deleted mice. *Nkx2-1* is not expressed within RP but is strongly expressed in multiple discreet regions of the brain, including the ventral diencephalon [14]. In *Nkx2-1*^*−/−*^ mutants, the infundibulum was absent [15] resulting in a fully penetrant phenotype, but the phenotype is not limited to the *Nkx2-1*-expressing tissues; all three lobes of the pituitary were also absent, highlighting a requirement for infundibular signals for proper RP induction.

Sonic hedgehog (SHH) pathway activation is necessary to induce specification of LHX3/LHX4 positive RP progenitors (Fig. 2). Genetic deletion of *Shh* from the anterior hypothalamus at 9.0 dpc, led to a failure to activate expression of *Lhx3* and *Lhx4* [16]. This resulted in a phenotype similar to *Lhx3*^*−/−*^*;Lhx4*^*−/−*^ double mutants with developmental arrest of RP and loss of tissue by 12.5 dpc. SHH alone however, is not sufficient to induce *Lhx3*/*Lhx4* expression. This process also requires BMP and FGF ligands to pattern the early pituitary and contribute to the positional determination of the mature endocrine cell types [17]. BMP4 was one of the first signals to be identified as driving the initial commitment of a fraction of the oral ectodermal cells to a pituitary cell fate. When the invaginating RP makes contact with the infundibulum, this upregulates expression of *Bmp4* [18, 19]. Homozygous null *Bmp4*^−/−^ mice display a failure of RP invagination [14], confirming a requirement for this secreted ligand in RP. Additionally, misexpression of Noggin, a potent antagonist of BMP4 from the oral ectoderm, resulted in early developmental arrest of RP [18].

While the infundibulum is supplying BMP4, it also expresses FGF8 and FGF10 ligands (Fig. 2). Addition of FGF8 in RP explant cultures is sufficient to induce *Lhx3* expression in the absence of the infundibulum [17]. This would indicate that FGF8 originating from the infundibulum is needed to initiate *Lhx3* expression in the dorsal pouch, which is required for anterior pituitary specification and development. Misexpression of FGF8 in the ventral RP, driven by regulatory elements of *Cga* (encoding αGSU, the alpha subunit, common to LH, FSH and TSH), causes a patterning defect with repression of ventral fates, consistent with a role in cell specification [18]. Further evidence for the essential role of FGF signalling comes from studies on FGFR2. Two splice variants of FGFR2 exist, encoding two receptor isoforms with differential ligand specificities. Within the pituitary, FGFR2 IIIb is regarded as the most important of the two isoforms. IIIb is found to be epithelial while IIIc is primarily located in the mesenchyme [26]. By disrupting the FGFR2 IIIb isoform using gene targeting strategies, mice were generated with mutant FGFR2, incapable of signalling through binding of FGF 1, 3, 7, and 10. In the FGFR2 IIIb-null mutant mouse, RP is completely absent by 14.5 dpc due to rapidly undergoing apoptosis, thus highlighting the importance of FGF signalling in maintaining cell survival [26]. In comparison, null mouse mutants of FGFR2 IIIc, which is restricted to the mesenchyme, are both viable and fertile, but exhibit proportional dwarfism. Despite this phenotype, analyses of pituitary morphology or function were not undertaken [27].

By acting on the as-of-yet uncommitted ectodermal progenitors, diencephalic-derived SHH, FGFs and BMP4 induce *Lhx3* expression. FGFs from the infundibulum further drive the dorsal cell proliferation, while SHH promotes proliferation of periluminal RP progenitors [16]. Meanwhile, as outlined below, ventral expression of BMP2 induces ventrally-restricted genes such as *Isl-1* and *Cga* [17]; while RP is receiving BMP4, FGF8 and FGF10 from the infundibulum, initiating patterning events, a second signalling centre activates ventrally in RP. From 9.5 dpc, *Bmp2* expression is detected at the most ventral side of the invaginating RP (Fig. 2). Expression at this stage is confined by a boundary demarcated by *Shh* expression in the oral ectoderm [18]. BMP2 expression continues to expand throughout the pouch and only ceases at 12.5 dpc, as a consequence of Chordin expression in the caudal mesenchyme. Chordin is a BMP2 & BMP4 antagonist whose expression facilitates maintaining a ventrodorsal BMP2 gradient [18]. Evidence for the importance of BMP2 in maintaining and specifying ventral pituitary cell types comes from in vivo overexpression models. When *Bmp2* was placed under the control of a *Cga* regulatory region to drive expression in αGSU positive cells, this led to a loss of TSH, PRL and GH expression. Indicating that appropriate downregulation of BMP2 needs to occur for terminal differentiation of these cell types.

While the precise location of originating WNTs in the pituitary primordia is an area of intense debate, WNT signalling itself is a vital contributor when it comes to appropriate specification of cell types. Multiple pituitary abnormalities have been observed by genetically disrupting *Wnt5a* and *Wnt4* [18, 28]*. Wnt4* is expressed from 9.5 dpc and by 10.5 dpc, WNT5A protein can be detected in RP in wild type embryos [29]. By 11.5 dpc, expression of these factors is detectable both in the rostral and caudal domains of the ventral diencephalon [30], overlapping with a major TCF7L2 expression domain which is induced via WNT [30, 31]. Analysis of *Wnt5a*^*−/−*^ as well as *Wnt4*^*−/−*^*;Wnt5a*^*−/−*^ double mutants revealed expanded FGF10 and BMP4 infundibular domains were responsible for pituitary dysmorphology [30]. This suggests that WNT signalling represses FGF and BMP signalling in the infundibulum. Additionally, single and double mutants exhibit depressed POU1F1 expression, leading to a mild anterior lobe hypoplasia. This is in part due to the failure of progenitors to proliferate and appropriately differentiate into the three POU1F1-dependent hormone producing cell types [30]. Olson et al*.* (2006) showed that β-catenin, a key effector of WNT signalling, directly interacts with PROP1 during pituitary organogenesis, leading to the activation of *Pou1f1* [29]*.* Therefore, WNT signalling is essential for POU1F1 lineage specification.

In addition to signals originating from the infundibulum, the extracellular matrix (ECM) plays an important part in pituitary organogenesis [32]. The ECM is a large network of proteins and other macromolecules, essential for cellular support, adhesion, migration, and propagation or sequestration of signals, integral to stem cell niches [33, 34]. ECM components including laminin, fibronectin and collagen IV have been shown to be present in and around the epithelial cells of RP in Syrian hamsters [35]. More recently it was shown that the laminin composition of the rat RP, differed from the epithelial stem cells of the marginal zone postnatally [36], indicating that remodelling of ECM proteins may be important for the maturing stem/progenitor cells over time. Furthermore, ECM components (laminin, collagen I and collagen IV) have been shown to influence *Pomc* transcription [37], suggesting that they actively play a role in the regulation of endocrine cell function in both physiological and pathological situations. ECM influence on the pituitary stem cell niche is yet to be investigated thoroughly.

## The function of stem cells in the anterior pituitary

**Fig. 3:**
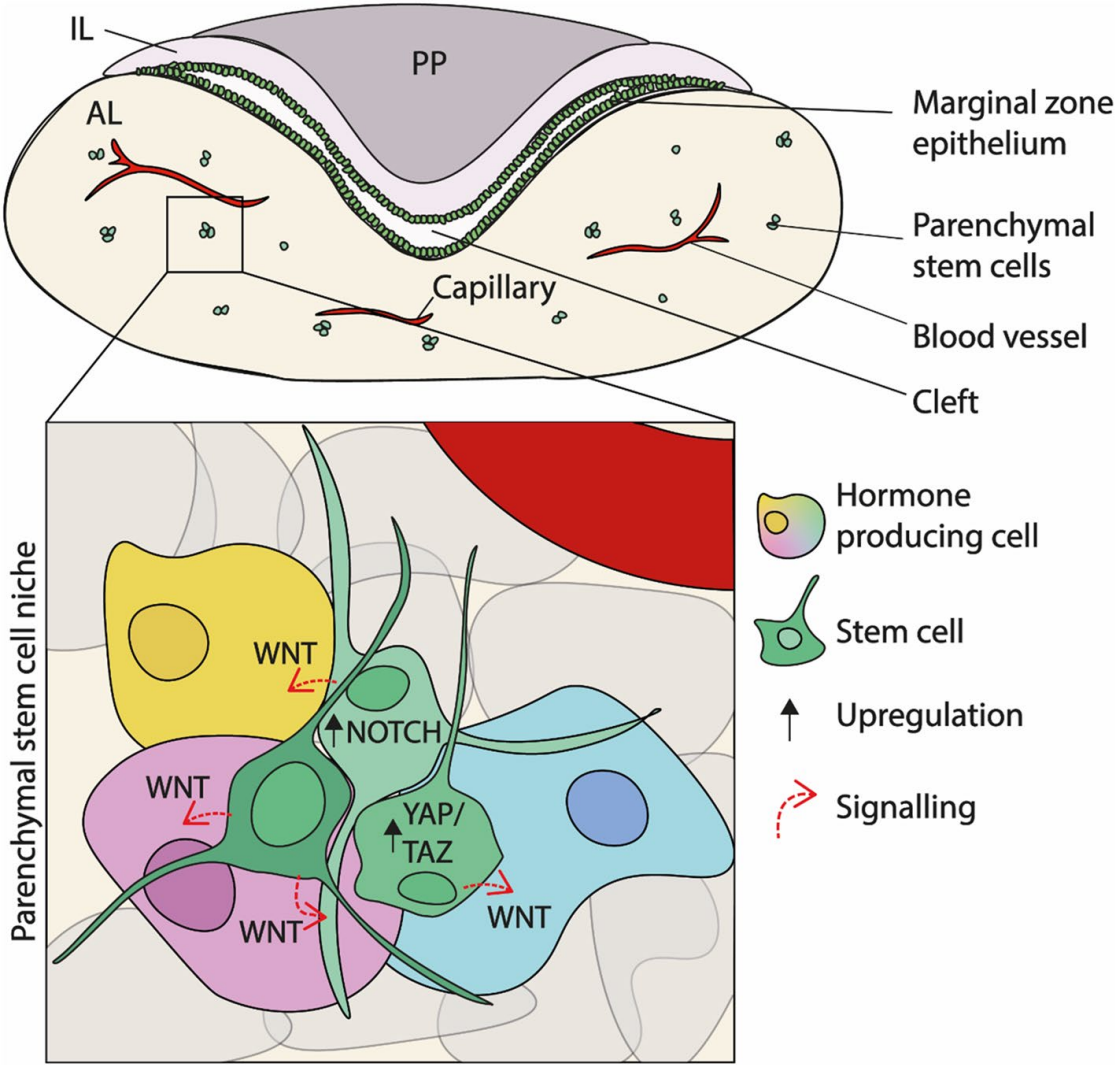
The postnatal pituitary stem cell niche. Stem cells (green) are present in the marginal zone cleft, lining the anterior and intermediate lobes, as well as in the parenchyma of the anterior pituitary. The stem cells are present within a niche, where they maintain high intracellular Notch and YAP/TAZ signalling. They secrete factors including WNT ligands, which are perceived by surrounding cells undergoing commitment, to promote their proliferation and expansion. Extrinsic signals important for pituitary stem cell maintenance and function are poorly defined. *PP* posterior pituitary, *AL* anterior lobe, *IL* intermediate lobe.

The first indication that stem cells were present in the adult pituitary came from cell culture experiments indicating an expanding cell type, where a population of cells not expressing hormones had the capability to form adherent colonies [38]. This population included S100β-expressing cells, described as folliculostellate cells of the anterior pituitary [39], and were able to uptake β‐Ala‐Lys‐N ε AMCA (AMCA is 7‐amino‐4‐methylcourmarin‐3‐acetic acid) and enabled isolation of this population using a fluorescent substrate. In parallel, cells expressing *Sca1*, *Oct4* and *Nanog* were shown to be capable of expanding as non-adherent spheres [40]. Amongst these are a population that can efflux Hoechst 33342, generating a very distinctive side scatter profile upon flow sorting [40]. The population of cells with these characteristics, was shown to express stemness markers such as SOX2 and SOX9 [41]. Using genetic tracing experiments it has been identified that these SOX2 positive cells (the majority of which subsequently express SOX9) are *bona fide* stem cells of the anterior pituitary and these can differentiate into all mature hormone-producing lineages and contribute to organ homeostasis during early postnatal and adult life [1, 2]. SOX2 positive cells are present within the postnatal pituitary in a single layered epithelium (or marginal zone) between the IL and the AL of the AP, and as clusters within the parenchyma of the AL [1, 2] (Figs. 1, 3).

While the pituitary of neonates contains all the hormone-producing cell types, the gland continues to expand in size during the postnatal growth period and into adulthood. In mice, after postnatal day 21 (P21), anterior pituitary expansion progresses slowly, and no major cell remodelling takes place unless there are specific physiological challenges. The cellular composition of the gland changes in challenging states such as pregnancy and lactation, with an associated expansion of the endocrine cell types required to drive an appropriate physiological response e.g. lactotrophs in this example, alongside an increase in lactotroph size. Experimental evidence that the adult pituitary can regenerate following injury came from studies whereby an inducible diphtheria toxin receptor gene was placed under the control of Cre recombinase, driven by growth hormone regulatory sequences (transgenic *Gh-Cre* mouse), i.e. expressed once *Gh* is expressed and in *Gh*-expressing cell descendants. Since mice do not normally express the diptheria toxin receptor, administration of diptheria toxin selective kills somatotrophs. As expected, the resulting *Gh-Cre;R26*^*iDTR/*+^ mice treated with diphtheria toxin showed dramatic (but not entire) ablation of somatotrophs [42]. In a similar study targeting lactotrophs, a *Prl-Cre;R26*^*iDTR/*+^ transgenic mouse was constructed whereby diphtheria toxin administration resulted in ablation of lactotrophs [43]. In both models, the remaining compartments recovered the cells which were lost, thereby indicating the regenerative capacity of the pituitary and hinted at the existence of putative stem or progenitor cells capable of regenerating the injured gland. This regeneration could have resulted from the PSCs, from POU1F1+ lineage-committed progenitor that are still not terminally differentiated, or by remaining GH+ or PRL+ cells themselves. It is important to note that this regenerative capacity was also shown to dwindle with age, with the highest response seen in young animals [44]. Both studies made the observation that the epithelial SOX2+ marginal zone layer appeared expanded in response to injury, although it was not the only source of new hormone cells, since cells from across all committed lineages have also been shown to enter cell cycle [44]. It is of ongoing interest to identify the molecular mechanisms that are governing pituitary stem cell properties such as self-renewal and commitment in the context of both normal growth, and response to physiological challenge.

## Signalling in the postnatal pituitary stem cell niche

Within the postnatal gland, hormone-producing cells exist in three-dimensional networks of differentiated cells allowing co-ordination for hormone secretion and response to alteration in hormone requirements (Fig. 1). Additionally, hormone-producing cells organise in preferred zones within the pituitary as well as adjacent to capillaries, required for hormone secretion into the blood stream. As such, GH cells form strands of connected cells close to capillaries, and the network displays sex differences between males and females, the most striking in pubertal males. At this time, GH cells increase clustering around strands, permitting large increases in GH secretion in response to the same stimulatory GHRH signal compared to females, an effect which is lost when cells are isolated [45]. In contrast, PRL cells form honeycomb-like structures and during lactation cells increase contact and size, to increase PRL hormone production upon suckling. These effects have been shown to last months after lactation and this organisational rearrangement even results in improved PRL secretion when nursing future litters [46]. Alternately, ACTH+ cell bodies are typically observed at a distance from each other and blood vessels, however, they possess long cytoplasmic projections (cytonemes) which connect to other ACTH cells and to capillaries, allowing direct hormone secretion [45].

Connected stem cell networks have similarly been observed, as SOX2+ cells also possess long cell protrusions which connect to other SOX2+ cells and hormone-producing cells within the gland, both from the marginal zone epithelium and within the parenchyma [47] (Fig. 1, Fig. 3). Previously termed folliculostellate cells, PSCs have also been shown to form an excitable network, which can transmit a Ca^2+^ signal long range across the gland between cells, connecting via gap junctions that also permit transmission of signalling molecules [48]. Such signals have been posited to control and co-ordinate hormone release from different parts of the organ. Work is ongoing to understand how structural networks permit hormone co-ordination and control, although communication between cells via signalling pathways has also been explored within the gland to better understand cell dynamics. The importance of studying and deciphering cell–cell signals across the pituitary stem cell niche stretches beyond basic biological understanding, and into physiological and clinical implications. All endocrine populations of the pituitary gland are capable of responding to physiological demand and are postulated to have the capacity to give rise to tumours [49]. If signals originating from, or mediated by, PSCs are implicated in the functional control of endocrine populations in humans, then PSCs are also likely to be implicated in normal physiological responses and disease. Therefore targeting PSCs can be central to modulating cellular behaviour of diverse cell types, in a translational or clinical setting.

Multiple cell signalling pathways are likely to be involved in the complete maintenance of pituitary stem cells and their niche, therefore future research utilising un-biased approaches such as single cell sequencing may be key in fully understanding control. In recent years, using targeted mouse genetic approaches, three key signalling pathways have been shown to regulate self-renewal and differentiation of stem cells: YAP/TAZ, Notch and WNT.

Hippo signalling through YAP/TAZ has been shown to control size of multiple organs through regulation of proliferation, apoptosis and stem cell activity [50]. The Hippo pathway is an inhibitory kinase cascade, whereby MST1/2 kinases activate LATS1/2 kinases, which in turn phosphorylate and inactivate effectors YAP and TAZ (WWTR1) resulting in their cytoplasmic retention and degradation. When the Hippo pathway is inactive, YAP/TAZ enter the nucleus and with DNA binding partners TEADs, promote transcription of target genes. Within the pituitary, expression of effectors YAP and TAZ is seen in SOX2+PSCs both in mouse and human glands (Fig. 3), while presence of phosphorylated YAP confirms activity of kinases in the postnatal organ [7, 51]. Upstream regulation of Hippo signalling via MST kinases has been shown to be dispensable in the pituitary, however, inactivating mutation of the kinase LATS1, which directly inhibits the effectors, results in tumour formation, where these aggressive tumours are comprised of proliferating, YAP/TAZ-accumulating, SOX2+ cells [52]. Similarly, expression of a constitutively active form of YAP at early postnatal time points also increases proliferation of SOX2 cells, resulting in an increase in PSCs within the gland. However, this does not result in tumour formation [7], suggesting a dose-dependent control of stem cell activity. These additional SOX2 cells were also shown to be able to differentiate into all hormone lineages upon reversion of YAP overexpression, demonstrating that control of YAP acts to maintain the stem cell state. Furthermore, within the hormone-producing rat tumour cell line GH3, inhibition of *Lats1* by siRNA also reduced activation of the *Prl* and *Gh* promoters [53], such that YAP/TAZ effectors may be able to supress differentiation of hormone lineages, although the validation and precise mechanisms for this have not yet been identified. Both YAP and TAZ have been reported in other cell types in vitro to interact with the SWI/SNF class of ATP-dependent chromatin remodellers, both to promote and inhibit expression of transcriptional targets [54]. More investigation is needed into how YAP/TAZ and their downstream effectors, the TEADs, interact with chromatin to initiate or repress target gene transcription within the anterior pituitary.

No specific ligand or receptor has been implicated in the Hippo pathway, with upstream regulation being linked to mechanical signalling: increased nuclear localisation of YAP/TAZ is seen in cells plated on a stiff matrix, upon sparse cell plating and within stretched cells [55]; and GPCRs (often acting through Rho/Actin) [55], which have not been confirmed within the pituitary. Apical protein NF2 (Merlin) and AMOTs have also been linked to upstream regulation of LATS activity [56]. Additionally, in *Drosophila* and some mammalian tissues, the Hippo pathway can be regulated via the atypical protocadherins Fat and Dachsous [56], although within the pituitary, mutations in FAT4 and DCHS1/DCHS2 do not appear to influence YAP/TAZ function [57]. Extracellular control of the Hippo pathway within the pituitary remains to be elucidated.

Notch signalling in the postnatal pituitary has also been linked to PSC regulation; expression of NOTCH2/NOTCH3 receptors and ligands JAG1 and DLL1 has been shown in embryonic progenitors and in postnatal PSCs [58–61]. Ligands DLL1 and DLL3 are also expressed in committed cells, as well as DLK1, which is detected in GH+ somatotrophs in humans, and reported in somatotrophs, lactotrophs and thyrotrophs in mouse [62, 63], indicating an additional role in differentiated cells. Alongside this, the Notch-associated transcription factor GRHL2 also marks pituitary stem cells, which has been identified as a downstream target of Notch signalling. GRHL2 has been linked to inhibition of EMT, maintenance of a progenitor phenotype and control of differentiation in other organs, including the lung and pancreas [64, 65] providing a further link between Notch signalling and the stem cell state.

Reduction of Notch signalling through loss of NOTCH2 [66], effector RBPJ [51] and targets HES1 and HEY1 result in reduction of the stem cell pool [7, 53, 66], which supports a role for Notch signalling in the maintenance of an undifferentiated, proliferative stem cell state. While most differentiated cells appear insensitive to changes in Notch signalling, HES1 has been shown to play a role in melanotroph specification, where cells lacking *Hes1* in the developing embryo differentiate into somatotrophs [7].

Cells across all lineages in the postnatal anterior pituitary, including SOX2+ stem cells, activate the WNT pathway postnatally, which remains important to promote cell proliferation, in addition to its role in the specification of the POU1F1-lineage. Genetic fate mapping of cells that activate the pathway at early postnatal stages (expressing WNT target *Axin2*) show that the majority of new pituitary cells are derived from AXIN2+ cells [67]. Surprisingly, the source of key WNT ligands in the postnatal gland was shown to be pituitary stem cells (Fig. 3). Binding of the 19 different WNT ligands to Frizzled (Fzd) receptors can be quite promiscuous and often with varying outcomes in downstream component activation, depending on the ligand-receptor pair [68]. All 10 *Fzd* receptor genes are expressed in the postnatal mouse pituitary gland, with stem cells most highly expressing *Fzd1*, *Fzd3*, *Fzd4*, *Fzd6* and *Fzd7* [67]. It has therefore been challenging to identify the key ligand-receptor pairs responsible for signalling events in the pituitary gland. Global genetic inhibition of WNT secretion, exclusively within the SOX2+ cell compartment, leads to a reduction of proliferation in both PSC and hormone-producing cell populations. As such, WNT signalling is required for autocrine signalling among PSCs as well as in a paracrine manner to promote proliferation in committed progenitors and/or hormone-producing cells. This previously undescribed function for stem cells, acting as instigators of proliferation of neighbouring cells, remains to be shown in other organs. However, within the pituitary, where the majority of new cells during the postnatal period are not generated from stem cells, this appears to be an integral active role for the stem cell compartment, in addition to acting as a reserve source of new cells.

Additional understudied signalling pathways are likely to be involved in maintenance of pituitary stem cells. There have been no compelling studies from mouse, that analyse the postnatal role of FGFs or BMPs within the anterior pituitary, despite emerging data from sequencing studies that ligands continue to be expressed after morphogenesis and into adulthood [69, 70]. BMPR1A is detected in mouse gonadotroph cell lines and enables signalling of BMP2 [71], however efficient murine genetic deletion of *Bmpr1a* in gonadotrophs did not result in a phenotype [72]. Mutations activating the MAPK signalling pathway result in tumour formation, showing an increase in proliferation of SOX2+ cells and impairment of differentiation [73]. Ephs and Ephrins are also expressed in the pituitary and within PSCs: ligand EPHA4A is expressed in GH cells, posterior lobe and endothelial cells; EPHB1 is seen in gonadotrophs; EPHB2 in corticotrophs, while EPHB3 and receptor EphrinB2 are seen in PSCs [74–76]. Such expression suggests signalling from PSCs to populations of differentiated cells as well as amongst stem cells themselves, but the effects of Eph/Ephrin signalling have not been confirmed. Further non-biased approaches may be able identify novel pathways that are important in the pituitary allowing for subsequent functional exploration.

## Cytokines and growth factors in the pituitary gland

Aside from signalling pathways, the role of cytokines and growth factors have also been explored in pituitary development, more closely reviewed in [77–79]. Leukemia inhibitory factor (LIF) and LIF receptor (LIFR) are both expressed in the anterior pituitary and have been strongly implicated in regulation of corticotrophs in the context of the hypothalamic–pituitary–adrenal axis. In human fetal pituitaries, LIF expression has been confirmed in ACTH+ , GH+ and non-hormone-expressing cells [80]. *Lif*^*−/−*^ null mutants have reduced ACTH secretion and LIF has been implicated in corticotroph differentiation [81, 82]. Similarly, LIFR knockout mice showed reduction in ACTH+ corticotrophs and dampened stimulation of the hypothalamic–pituitary–adrenal axis compared to control littermates [83]. Conversely, transgenic overexpression of LIF led to high levels of circulating ACTH and corticosterone [80, 84]. These overexpression models also report poor growth and smaller mice, typically associated with defects in the POU1F1 lineage (somatotrophs) [80, 84], which is unsurprising since expression of LIF was shown in GH+ somatotrophs during human development, but the underpinnings of this phenotype were not characterised in the mouse models. Intriguingly, both murine studies also report formation of cysts resembling Rathke’s cleft cyst (RCC), with LIF expression confirmed in cells lining cysts within human samples of RCC. Since an RCC-like phenotype was also reported in studies manipulating embryonic YAP levels in mouse, where the cysts were derived from the epithelial PSCs [85] or following deletion of *Isl1* [86], it would be interesting to determine if the LIF pathway normally interacts with either of these components during development. In LIF and LIFR mutant mice, an increase in other inflammatory signals were also identified, including IL6, IL1A and TNFα [82, 83].

Other growth factors have also been implicated in the control of hormone-expressing endocrine populations. Addition of nerve growth factor (NGF) to prolactinoma cells in vitro, appeared to promote maturation of PRL cells [87]. Expression of NGF and its receptor NTRK1 (TrkA) has been demonstrated in the pituitary gland, however their precise role has not been identified [87] [88].

Epidermal growth factor (EGF) expression has also been described in the pituitary within multiple hormone-producing cell types [89–91]. Transgenic overexpression of dominant negative mutation of the EGF receptor, EGFR, driven in somatotrophs, resulted in dwarfism and failed lactation in females. This is thought to be due to reduced numbers of GH+ and PRL+ cells [92], suggesting a role of EGF in differentiation within the PIT1 lineage. Subsequently, sequencing experiments have identified EGFR expression in stem cells [41, 93], but as for several of the growth factors likely to play a potential role, the function of EGF signalling in PSCs has not been investigated. Transforming growth factor alpha (TGFα), also binds EGFR, and similarly has been shown to be expressed by pituitary cells, however there is no consensus on the identity of cells expressing and secreting this factor [94–96]. Therefore, there is strong evidence that EGFR signalling has important roles in anterior pituitary cell function, but little knowledge of the mechanisms, meriting further study.

An example of immune-pituitary interactions has been identified in a mouse mutant of the chemokine receptor, CXCR2, where null mice with chronic systemic infections were seen to exhibit impaired reproductive ability in both males and females, an effect not seen in controls [97]. *Cxcr2*^*−/−*^ female mice had impaired development of ovaries and mammary glands, as well as lower levels of circulating FSH, LH, PRL and GH; however, these could be rescued by transplant of organs from WT controls. In comparison, mutants appeared normal when under pathogen-free housing conditions. This work highlights immunological stress as a potential modulator of pituitary activity.

Recent work has identified IL6 as a mediator of the differential regenerative response observed between the young and aged mouse pituitary. Vennekens et al. identified upregulation of IL6 in damaged young (8–12 week) pituitaries but not in aged (10–15 month) organs, correlating with regenerative ability seen in these models [98]. Aged pituitaries were also found to express higher than normal levels of *Il6* compared to younger cohorts, alongside increased inflammatory gene and pathway expression. Furthermore, IL6 could be used in vitro to stimulate proliferation of pituitary stem cells in both ages [98], suggesting that the in vivo environment also plays a role in PSC response. Since IL6 is a component of the senescence-associated secretory phenotype (SASP), the accumulation of senescent cells from the immune or pituitary compartments with age, including PSCs, may contribute to this phenotype. IL6 can have distinct effects on cells, both in promoting population expansion as well as promoting cellular senescence [99], hence it is an important component both in normal pituitary regulation as well as the formation of tumours.

Modulation of the inflammatory response is an important step in the development of the childhood pituitary tumour adamantinomatous craniopharyngioma (ACP). The mouse models of ACP target a degradation resistant form of β-catenin to SOX2+ cells embryonically or postnatally. Instigated by expression of this mutation, these cells become senescent, exiting the cell cycle and initiating the SASP, secreting increased levels of growth factors, including *Shh, Bmp’s, Fgf’s, Cxcr4, Cxcl1, Ccl20, Tgfb, Il6, Il1a* and *Il1b.* Expression of SASP components leads to transformation of surrounding non-mutant cells, which end up forming the tumour mass, rather than the tumour originating from mutant cells i.e. paracrine tumour formation [1, 47, 100, 101]. Research also showed that tumourigenesis was reduced in older mice, which showed decreased senescence and SASP response [101]. As well as promoting tumour growth, SASP is thought to cause remodelling of ECM, which is more beneficial to tumour growth, and modulate the immune system preventing recognition, increasing tumour longevity.

Work utilising current knowledge of stem cells and differentiation hierarchy, alongside more recent techniques such as sequencing, may prove useful in further identifying the roles and requirements for cytokines and soluble growth factors for the whole tissue. These factors may provide useful tools in our ability to generate hormone producing cells in vitro and in vivo*.* Alongside this, knowledge of immunological and pituitary interactions will assist our understanding of normal homeostasis, as well as tumour development and progression, with the goal of developing better treatment options.

## Beyond signalling, towards cell behaviour

While cell-to-cell signalling is an integral component of niche dynamics and cell communication, it is the TFs at the end of a signalling cascade that ultimately drive cell behaviour by enforcing the necessary gene expression networks. These programs can broadly be classed as ‘behavioural’ or ‘identity’ programs, depending if they are non-cell type specific behaviours (e.g. mitosis), or integral to a cell’s identity (e.g. differentiation) [102].

A new classification of TFs has emerged whereby they can be termed ‘pioneer’ if able to modulate local condensed chromatin structure to gain access to otherwise inaccessible genomic regions. Pioneer TFs, either alone or together with co-factors, unwind heterochromatin revealing underlying specific binding motifs (reviewed extensively by Mayran et al. [103]). An example in the pituitary gland is PAX7, which controls fate specification of melanotrophs at the expense of corticotroph fate [104, 105]. Activation of PAX7 remodels unique enhancers, which allow TBX19 to bind [105]. Furthermore, it has been shown that even transient expression of PAX7 is capable of remodelling a unique combination of enhancers that are sufficient to drive the melanotroph cell fate. Further study is required to identify additional pioneer factors within the anterior pituitary gland, which are capable of uniquely specifying distinct endocrine cell types such as somatotrophs, lactotrophs or thyrotrophs, all of which are specified through the actions of POU1F1 via understudied downstream mechanisms.

## Deciphering anterior pituitary signalling centres using single cell technologies

Recent advancements in single cell sequencing technologies, such as single cell RNA sequencing (scRNAseq), have provided valuable insights into the cellular composition, detailed transcriptomics and complex networks within a diverse cohort of mouse and human tissues [70, 106–108]. As well as unearthing cellular heterogeneity, from these types of data, we can infer cell-crosstalk based on transcript expression of known receptor- and ligand- pairs. Analysis of these pairs throughout whole tissues was previously unachievable. In addition, the advent of single cell (or single nuclei) ATAC-seq has revolutionised the detail by which we can interrogate chromatin regulatory landscapes by measuring the accessibility at genomic loci in single cells [109]. This approach can be leveraged to identify novel pioneer factors controlling cell type commitment.

Over the last few years, multiple groups have utilised scRNAseq methods to scrutinise the transcriptome of the pituitary across various vertebrate species (Table 1), with the frequent emergence of new studies as the technologies become established. For a comprehensive review see [110].

**Table 1:** Details of scRNAseq studies on in vivo pituitaries to date

Authors	Pituitary information	Cell numbers and additional information
Cheung et al*.* [111]	Whole mouse pituitary	13,663 cells from 6 × 7 week male mice
Fletcher et al*.* [112]	Rat anterior pituitary	6896 cells from 2 male and 2 female postpubertal rats
Mayran et al*.* [103]	Mouse anterior pituitary	9269 cells from 1 male 4 month old mouse
Cheung et al*.* [110]	Whole mouse pituitary	6492 cells from 1 × P4 female mutant mouse 6705 cells from 1 × P4 female control mouse
Chen et al*.* [113]	Mouse intermediate and posterior pituitary lobes	528 cells from 1 × 3 month old male mouse
Ho et al*.* [114]	Whole mouse pituitary	3532 cells from 1 × 13 week old virgin female 7614 cells from 2 × 13 week old lactating females 7443 cells from 4 × 8 week old male and females 2596 cells from 8 week old acromegalic female
Zhang et al*.* [108]	Human fetal pituitary	4113 cells from 21 human fetuses
Lopez J.P et al*.* [115]	Whole mouse pituitary	9,879 cells from 10 week-old male mice: 5 x unstressed and 5 x chronically stressed
Moncho-Amor et al. [116]	Intermediate lobes of control and mutant mouse pituitaries	2110 cells from 3 month old control male and female mice 8832 cells from 3 month old mutant male and female mice
Cui et al*.* [117]	Human neuroendocrine tumours	2679 cells from 21 human patients
Ruf-Zamojski et al. [70]	Whole mouse pituitary	35,707 nuclei from 6 × 10–12 week old male and female mice
Allensworth-James M et al*.* [118]	Whole mouse pituitary	18,301 cells from 6 x 8-week old diestrous female mice
Vennekens et al*.* [98]	Anterior pituitaries of control and mutant mice	7977 cells from 9–11 weeks old control male mice 7000 cells from 9–11 weeks old mutant male mice 5278 cells from 14 month old control male mice 5860 cells from 14 month old mutant male mice
Zhang et al*.* [119]	Whole chicken anterior pituitary	6733 cells from 6 male chicken and 9,919 cells from 6 female chicken both at 12 months
Siddique et al*.* [120]	Whole adult teleost pituitaries	2592 cells from female teleost and 3804 cells from male teleost
Fletcher et al*.* [131]	Whole rat pituitary	15,876 cells from 60 × 75 day -old female rats (Combined from; separated rat anterior pituitary, separated rat intermediate lobe and posterior pituitary, and whole rat pituitary)
Zhang et al*.* [121]	Human pediatric, adult and aged pituitary	76,016 nuclei for snRNAseq 15,024 nuclei in the same-cell sn multiome (ATAC and RNA)
Laporte E et al. [122]	Anterior pituitaries of control and mutant mice	9618 cells from control 2 × P7 mice 11,801 from mutant 2 × P7 mice

The first of these in vivo pituitary cell studies, carried out on 7 week old male mice by Cheung et al*.* [111], highlighted new markers for several cell types, including *Cyp2f2, Lcn2, Aldh1a2* and *Rbpms* for stem cells. This study paved the way for an onslaught of pituitary scRNAseq investigations, including another from the same group in 2020 highlighting the importance of previously described stem cell marker, *Aldh1a2* [69], encoding an enzyme crucial for Retinoic Acid (RA) synthesis*.* RA signalling is known to be important for proper hormone production among the *Pou1f1* lineage cell types [123–125] and is thought to be produced, in part, by the PSCs [126]. Concordant with this, Cheung et al. show, through scRNAseq of embryonic conditional knock-out of *Aldh1a2* in pituitary progenitors, with subsequent reduction in RA, that RA signalling and *Aldh1a2* are both crucial for proper pituitary formation and lineage stratification. These data highlight one way that scRNAseq analyses can be used to inform and resolve PSC-related signalling events crucial for the homeostasis of the gland [69].

Additional new generation sequencing techniques also add depth to our knowledge of cellular interactions within the PSC niche. For example, recent single nuclei RNAseq (snRNAseq) and snATACseq profiling data from adult male and female mice gave a first look into regulatory events coordinating the function of the murine pituitary [70]. Two stem cell clusters were distinguishable, both sharing many of the same PSC markers as previously described, including *Rbpms* and the YAP/TAZ signalling pathway readout *Cyr61*, however one is delimited by a drop in expression within the shared markers (e.g. *Grin2a*)*.* Utilising the multimodal datasets, the authors used PLIER methodology to find latent variables (LVs), modules of genes that vary across cell types/samples, taking into account coordinated gene expression and accessible chromatin regions. LVs in both the RNAseq and ATACseq data highlighted genes including *Aldh1a2*, *Krt18* and *Pax6* as key players in PSC identity and pathway analysis of the full LV lists pulled out functions such as ‘Cytokine Receptor Interaction’ and ‘ECM Receptor Interaction’. From this study we find that paired sequencing data, integrating different sequencing readouts, can implicate and confirm important cell signalling patterns associated with PSCs and their surrounding niche [70].

Currently, it is unclear by which mechanisms PSCs reactivate in response to organ damage, and how this reaction changes during aging. In an attempt to address this, Vennekens and colleagues used scRNA sequencing of an inducible mouse model that conditionally ablates somatotrophs and lactotrophs to assess PSC reactivation upon insult. Building upon this previously described *Gh-Cre*/*R26*^*iDTR/*+^ model [42], they found that prior to somatotroph replacement, there was an acute increase in PSC proliferation in young adult mice, which declined from 10 to 15 weeks of age. Additionally, the authors found that *Il6*, a pro-inflammatory cytokine, was upregulated in the young pituitary PSCs after tissue injury but this did not occur in aged mice. Not only does this study deepen our understanding of GH+ and PRL+ cell-PSC crosstalk, but it also reinforces the need for a more thorough investigation of the complex nature of immune-PSC interactions over a range of ages [98].

## Epithelial—mesenchymal interactions in the pituitary stem cell niche

In a pioneering study from Zhang et al*.* [108], cells from human pituitaries across a range of embryonic ages were isolated and subjected to single cell RNAseq [108]. The subsequent analysis identified PSCs with a similar transcriptomic fingerprint as previously described, consisting of PSC markers such as *SOX2*, *YAP1*, and *HES1*. In addition to resolving differentiation trajectories, the authors took advantage of the abundance of collagen-expressing mesenchymal cells to run a secondary enrichment analysis. This identified that human fetal PSCs were enriched for gene members of pathways such as ‘tight junctions’, and ‘ECM interactions’. The two populations were then examined for potential receptor-ligand interactions, which implicated multiple key signalling pathways such as Notch and Ephrin as mediums of communication between PSCs and mesenchymal cells. Further histological validation confirmed that a subset of human SOX2-expressing PSCs are encompassed by collagen III-expressing mesenchymal cells, indicating that direct cell-to-cell contact may be playing a role in bidirectional signalling between these two cell types [108]. Based on their findings, the authors suggest that WNT, BMP, Eph/Ephrin and Notch signalling may be involved in communication between mesenchymal cells and PSCs, but this has not yet been experimentally investigated.

Single cell RNA-seq data can not only be used to describe enrichment of specific transcripts within heterogeneous cell populations, during homeostasis and after challenge. It can also be used to infer cell-to-cell communication through enrichment analyses of known ligand-receptor pair transcripts. Analysis packages such as CellPhoneDB [127], NicheNet [128], SingleCellSignalR [129], and CellChat [130] utilise known ligand-receptor interactions, including co-factors and co-receptors, to visualise intercellular communications from scRNA-seq data. These analyses can elucidate new signalling pathways involved in PSC-niche maintenance and provide clues on areas for future study. They also enable assessment of interactions between PSCs and other supporting cells such as endothelial cells and connective tissue. In a recent report, Fletcher et al*.* [131] use CellChat to investigate WNT, BMP, Notch, and FGF signalling pathway interactions between the pituitary stem cells (referred to as folliculostellate cells), pericytes, endothelial cells and pituicytes in the postnatal rat pituitary. Interestingly they find *Bmp2* and *Bmp4,* expressed by pericytes and endothelial cells, respectively, with their receptor transcripts (*Bmpr1a* and *Bmpr2*) enriched within PSCs [131]. These results provide details on cross-talk mechanisms between PSCs and their surrounding niche, which could be further supported by spatial validation at the protein level. It would be interesting to undertake further CellChat analyses at various ages to elucidate the PSC niche in detail and how this may be adapted throughout life.

## Considerations and future directions

As summarised, scRNAseq provides unprecedented power in allowing us to infer signalling events and communication dynamics, between cell types within a given tissue. This can provide valuable insights into networks and formation of hypotheses around cell-to-cell interactions. Currently, a limiting factor of droplet-based technologies is that cell interactions can only be inferred, as dissociation of the original tissue source into single cells is required prior to sequencing. Accordingly, up-and-coming technologies are aiming to preserve tissue composition while extracting the mRNA to reconstruct the underlying transcriptome. To gain deeper insight into the cellular networks within the whole pituitary, it would be of key interest to undertake in situ sequencing. Commercially available products allow imaging of tissue slices which are overlayed over spots that can capture mRNA upon permeabilisation of the tissue. A limitation of these methodologies is that due to the spacing of capturing spots, multiple cells can overlap over the same region, thereby giving a mixed mRNA signal consisting of transcripts from multiple cells. For many tissues with similar cells grouped in distinct locations, such as in the brain, these methods can provide useful information. However, as discussed, the pituitary gland consists of compartments that house many mixed cell types, therefore for insightful information from in situ sequencing, a higher resolution, at a spot-diameter equivalent to that of a nucleus would be necessary. Additionally, cellular networks and communication between stem cells should be scrutinised by snRNAseq after isolation and enrichment to obtain sufficient information. Coupled with thorough functional validation, in the coming years, these approaches should identify and elucidate novel uni- and multi-directional mechanisms of cell communication and control in the pituitary stem cell niche.

## Data Availability

Not applicable.

## References

[1] Andoniadou CL, Matsushima D, Mousavy Gharavy SN, Signore M, Mackintosh AI, Schaeffer M, Gaston-Massuet C, Mollard P, Jacques TS, Le Tissier P, Dattani MT, Pevny LH, Martinez-Barbera JP (2013). Sox2(+) stem/progenitor cells in the adult mouse pituitary support organ homeostasis and have tumor-inducing potential. Cell Stem Cell.

[2] Rizzoti K, Akiyama H, Lovell-Badge R (2013). Mobilized adult pituitary stem cells contribute to endocrine regeneration in response to physiological demand. Cell Stem Cell.

[3] Gehart H, Clevers H (2019). Tales from the crypt: new insights into intestinal stem cells. Nat Rev Gastroenterol Hepatol.

[4] Kopp JL, Grompe M, Sander M (2016). Stem cells versus plasticity in liver and pancreas regeneration. Nat Cell Biol.

[5] Watt FM, Hogan BL (2000). Out of Eden: stem cells and their niches. Science.

[6] Baylis PH (1983). Posterior pituitary function in health and disease. Clin Endocrinol Metab.

[7] Raetzman LT, Cai JX, Camper SA (2007). Hes1 is required for pituitary growth and melanotrope specification. Dev Biol.

[8] Goldberg LB, Aujla PK, Raetzman LT (2011). Persistent expression of activated Notch inhibits corticotrope and melanotrope differentiation and results in dysfunction of the HPA axis. Dev Biol.

[9] Gage PJ, Suh H, Camper SA (1999). The bicoid-related Pitx gene family in development. Mamm Genome.

[10] Tremblay JJ, Lanctot C, Drouin J (1998). The pan-pituitary activator of transcription, Ptx1 (pituitary homeobox 1), acts in synergy with SF-1 and Pit1 and is an upstream regulator of the Lim-homeodomain gene Lim3/Lhx3. Mol Endocrinol.

[11] Szeto DP, Rodriguez-Esteban C, Ryan AK, O'Connell SM, Liu F, Kioussi C, Gleiberman AS, Izpisua-Belmonte JC, Rosenfeld MG (1999). Role of the Bicoid-related homeodomain factor Pitx1 in specifying hindlimb morphogenesis and pituitary development. Genes Dev.

[12] Suh H, Gage PJ, Drouin J, Camper SA (2002). Pitx2 is required at multiple stages of pituitary organogenesis: pituitary primordium formation and cell specification. Development.

[13] Dasen JS, Rosenfeld MG (1999). Signaling mechanisms in pituitary morphogenesis and cell fate determination. Curr Opin Cell Biol.

[14] Takuma N, Sheng HZ, Furuta Y, Ward JM, Sharma K, Hogan BL, Pfaff SL, Westphal H, Kimura S, Mahon KA (1998). Formation of Rathke's pouch requires dual induction from the diencephalon. Development.

[15] Kimura S, Hara Y, Pineau T, Fernandez-Salguero P, Fox CH, Ward JM, Gonzalez FJ (1996). The T/ebp null mouse: thyroid-specific enhancer-binding protein is essential for the organogenesis of the thyroid, lung, ventral forebrain, and pituitary. Genes Dev.

[16] Carreno G, Apps JR, Lodge EJ, Panousopoulos L, Haston S, Gonzalez-Meljem JM, Hahn H, Andoniadou CL, Martinez-Barbera JP (2017). Hypothalamic sonic hedgehog is required for cell specification and proliferation of LHX3/LHX4 pituitary embryonic precursors. Development.

[17] Ericson J, Norlin S, Jessell TM, Edlund T (1998). Integrated FGF and BMP signaling controls the progression of progenitor cell differentiation and the emergence of pattern in the embryonic anterior pituitary. Development.

[18] Treier M, Gleiberman AS, O'Connell SM, Szeto DP, McMahon JA, McMahon AP, Rosenfeld MG (1998). Multistep signaling requirements for pituitary organogenesis in vivo. Genes Dev.

[19] Rosenfeld MG, Briata P, Dasen J, Gleiberman AS, Kioussi C, Lin C, O'Connell SM, Ryan A, Szeto DP, Treier M (2000). Multistep signaling and transcriptional requirements for pituitary organogenesis in vivo. Recent Prog Horm Res.

[20] Susa T, Kato T, Yoshida S, Yako H, Higuchi M, Kato Y (2012). Paired-related homeodomain proteins Prx1 and Prx2 are expressed in embryonic pituitary stem/progenitor cells and may be involved in the early stage of pituitary differentiation. J Neuroendocrinol.

[21] Sheng HZ, Westphal H (1999). Early steps in pituitary organogenesis. Trends Genet.

[22] Kelberman D, Rizzoti K, Lovell-Badge R, Robinson IC, Dattani MT (2009). Genetic regulation of pituitary gland development in human and mouse. Endocr Rev.

[23] Dasen JS, Rosenfeld MG (2001). Signaling and transcriptional mechanisms in pituitary development. Annu Rev Neurosci.

[24] Sheng HZ, Zhadanov AB, Mosinger B, Fujii T, Bertuzzi S, Grinberg A, Lee EJ, Huang SP, Mahon KA, Westphal H (1996). Specification of pituitary cell lineages by the LIM homeobox gene Lhx3. Science.

[25] Sheng HZ, Moriyama K, Yamashita T, Li H, Potter SS, Mahon KA, Westphal H (1997). Multistep control of pituitary organogenesis. Science.

[26] De Moerlooze L, Spencer-Dene B, Revest JM, Hajihosseini M, Rosewell I, Dickson C (2000). An important role for the IIIb isoform of fibroblast growth factor receptor 2 (FGFR2) in mesenchymal-epithelial signalling during mouse organogenesis. Development.

[27] Eswarakumar VP, Monsonego-Ornan E, Pines M, Antonopoulou I, Morriss-Kay GM, Lonai P (2002). The IIIc alternative of Fgfr2 is a positive regulator of bone formation. Development.

[28] Cha KB, Douglas KR, Potok MA, Liang H, Jones SN, Camper SA (2004). WNT5A signaling affects pituitary gland shape. Mech Dev.

[29] Olson LE, Tollkuhn J, Scafoglio C, Krones A, Zhang J, Ohgi KA, Wu W, Taketo MM, Kemler R, Grosschedl R, Rose D, Li X, Rosenfeld MG (2006). Homeodomain-mediated beta-catenin-dependent switching events dictate cell-lineage determination. Cell.

[30] Potok MA, Cha KB, Hunt A, Brinkmeier ML, Leitges M, Kispert A, Camper SA (2008). WNT signaling affects gene expression in the ventral diencephalon and pituitary gland growth. Dev Dyn.

[31] Brinkmeier ML, Potok MA, Davis SW, Camper SA (2007). TCF4 deficiency expands ventral diencephalon signaling and increases induction of pituitary progenitors. Dev Biol.

[32] Paez-Pereda M, Kuchenbauer F, Arzt E, Stalla GK (2005). Regulation of pituitary hormones and cell proliferation by components of the extracellular matrix. Braz J Med Biol Res.

[33] Watt FM, Huck WTS (2013). Role of the extracellular matrix in regulating stem cell fate. Nat Rev Mol Cell Biol.

[34] Krivanek J, Soldatov RA, Kastriti ME, Chontorotzea T, Herdina AN, Petersen J, Szarowska B, Landova M, Matejova VK, Holla LI, Kuchler U, Zdrilic IV, Vijaykumar A, Balic A, Marangoni P, Klein OD, Neves VCM, Yianni V, Sharpe PT, Harkany T, Metscher BD, Bajenoff M, Mina M, Fried K, Kharchenko PV, Adameyko I (2020). Dental cell type atlas reveals stem and differentiated cell types in mouse and human teeth. Nat Commun.

[35] Horacek MJ, Thompson JC, Dada MO, Terracio L (1993). The extracellular matrix components laminin, fibronectin, and collagen IV are present among the epithelial cells forming Rathke’s pouch. Cells Tissues Organs.

[36] Ramadhani D, Tsukada T, Fujiwara K, Azuma M, Kikuchi M, Yashiro T (2014). Changes in laminin chain expression in pre- and postnatal rat pituitary gland. Acta Histochem Cytochem.

[37] Kuchenbauer F, Hopfner U, Stalla J, Arzt E, Stalla GK, Paez-Pereda M (2001). Extracellular matrix components regulate ACTH production and proliferation in corticotroph tumor cells. Mol Cell Endocrinol.

[38] Lepore DA, Roeszler K, Wagner J, Ross SA, Bauer K, Thomas PQ (2005). Identification and enrichment of colony-forming cells from the adult murine pituitary. Exp Cell Res.

[39] Vila-Porcile E (1972). Le rseau des cellules folliculo-stellaires et les follicules de l'adnohypophyse du rat (Pars distalis). Zeitschrift fr Zellforschung und Mikroskopische Anatomie.

[40] Chen J, Hersmus N, Van Duppen V, Caesens P, Denef C, Vankelecom H (2005). The adult pituitary contains a cell population displaying stem/progenitor cell and early embryonic characteristics. Endocrinology.

[41] Chen J, Gremeaux L, Fu Q, Liekens D, Van Laere S, Vankelecom H (2009). Pituitary progenitor cells tracked down by side population dissection. Stem Cells.

[42] Fu Q, Gremeaux L, Luque RM, Liekens D, Chen J, Buch T, Waisman A, Kineman R, Vankelecom H (2012). The adult pituitary shows stem/progenitor cell activation in response to injury and is capable of regeneration. Endocrinology.

[43] Fu Q, Vankelecom H (2012). Regenerative capacity of the adult pituitary: multiple mechanisms of lactotrope restoration after transgenic ablation. Stem Cells Dev.

[44] Willems C, Fu Q, Roose H, Mertens F, Cox B, Chen J, Vankelecom H (2016). Regeneration in the pituitary after cell-ablation injury: time-related aspects and molecular analysis. Endocrinology.

[45] Mollard P, Hodson DJ, Lafont C, Rizzoti K, Drouin J (2012). A tridimensional view of pituitary development and function. Trends Endocrinol Metab.

[46] Le Tissier PR, Hodson DJ, Lafont C, Fontanaud P, Schaeffer M, Mollard P (2012). Anterior pituitary cell networks. Front Neuroendocrinol.

[47] Andoniadou CL, Gaston-Massuet C, Reddy R, Schneider RP, Blasco MA, Le Tissier P, Jacques TS, Pevny LH, Dattani MT, Martinez-Barbera JP (2012). Identification of novel pathways involved in the pathogenesis of human adamantinomatous craniopharyngioma. Acta Neuropathol.

[48] Fauquier T, Guerineau NC, McKinney RA, Bauer K, Mollard P (2001). Folliculostellate cell network: a route for long-distance communication in the anterior pituitary. Proc Natl Acad Sci USA.

[49] Melmed S (2003). Mechanisms for pituitary tumorigenesis: the plastic pituitary. J Clin Invest.

[50] Yu FX, Zhao B, Guan KL (2015). Hippo pathway in organ size control, tissue homeostasis, and cancer. Cell.

[51] Zhu X, Tollkuhn J, Taylor H, Rosenfeld MG (2015). Notch-dependent pituitary SOX2(+) stem cells exhibit a timed functional extinction in regulation of the postnatal gland. Stem Cell Reports.

[52] Xekouki P, Lodge EJ, Matschke J, Santambrogio A, Apps JR, Sharif A, Jacques TS, Aylwin S, Prevot V, Li R, Flitsch J, Bornstein SR, Theodoropoulou M, Andoniadou CL (2019). Non-secreting pituitary tumours characterised by enhanced expression of YAP/TAZ. Endocr Relat Cancer.

[53] Kita A, Imayoshi I, Hojo M, Kitagawa M, Kokubu H, Ohsawa R, Ohtsuka T, Kageyama R, Hashimoto N (2007). Hes1 and Hes5 control the progenitor pool, intermediate lobe specification, and posterior lobe formation in the pituitary development. Mol Endocrinol.

[54] Hillmer RE, Link BA (2019). The Roles of Hippo Signaling Transducers Yap and Taz in Chromatin Remodeling. Cells.

[55] Dupont S (2016). Role of YAP/TAZ in cell-matrix adhesion-mediated signalling and mechanotransduction. Exp Cell Res.

[56] Zheng YG, Pan DJ (2019). The hippo signaling pathway in development and disease. Dev Cell.

[57] Lodge EJ, Xekouki P, Silva TS, Kochi C, Longui CA, Faucz FR, Santambrogio A, Mills JL, Pankratz N, Lane J, Sosnowska D, Hodgson T, Patist AL, Francis-West P, Helmbacher F, Stratakis CA, Andoniadou CL (2020). Requirement of FAT and DCHS protocadherins during hypothalamic-pituitary development. Jci Insight.

[58] Chen J, Crabbe A, Van Duppen V, Vankelecom H (2006). The notch signaling system is present in the postnatal pituitary: marked expression and regulatory activity in the newly discovered side population. Mol Endocrinol.

[59] Raetzman LT, Ross SA, Cook S, Dunwoodie SL, Camper SA, Thomas PQ (2004). Developmental regulation of Notch signaling genes in the embryonic pituitary: Prop1 deficiency affects Notch2 expression. Dev Biol.

[60] Zhu X, Zhang J, Tollkuhn J, Ohsawa R, Bresnick EH, Guillemot F, Kageyama R, Rosenfeld MG (2006). Sustained Notch signaling in progenitors is required for sequential emergence of distinct cell lineages during organogenesis. Genes Dev.

[61] Batchuluun K, Azuma M, Fujiwara K, Yashiro T, Kikuchi M (2017). Notch signaling and maintenance of SOX2 expression in rat anterior pituitary cells. Acta Histochem Cytochem.

[62] Cheung LY, Rizzoti K, Lovell-Badge R, Le Tissier PR (2013). Pituitary phenotypes of mice lacking the notch signalling ligand delta-like 1 homologue. J Neuroendocrinol.

[63] Scagliotti V, Esse R, Willis TL, Howard M, Carrus I, Lodge E, Andoniadou CL, Charalambous M (2021). Dynamic expression of imprinted genes in the developing and postnatal pituitary gland. Genes.

[64] Kersbergen A, Best SA, Dworkin S, Ah-Cann C, de Vries ME, Asselin-Labat ML, Ritchie ME, Jane SM, Sutherland KD (2018). Lung morphogenesis is orchestrated through Grainyhead-like 2 (Grhl2) transcriptional programs. Dev Biol.

[65] Nishino H, Takano S, Yoshitomi H, Suzuki K, Kagawa S, Shimazaki R, Shimizu H, Furukawa K, Miyazaki M, Ohtsuka M (2017). Grainyhead-like 2 (GRHL2) regulates epithelial plasticity in pancreatic cancer progression. Cancer Med.

[66] Nantie LB, Himes AD, Getz DR, Raetzman LT (2014). Notch signaling in postnatal pituitary expansion: proliferation, progenitors, and cell specification. Mol Endocrinol.

[67] Russell JP, Lim X, Santambrogio A, Yianni V, Kemkem Y, Wang B, Fish M, Haston S, Grabek A, Hallang S, Lodge EJ, Patist AL, Schedl A, Mollard P, Nusse R, Andoniadou CL (2020). Pituitary stem cells produce paracrine WNT signals to control the expansion of their descendant progenitor cells. Elife.

[68] Dijksterhuis JP, Baljinnyam B, Stanger K, Sercan HO, Ji Y, Andres O, Rubin JS, Hannoush RN, Schulte G (2015). Systematic Mapping of WNT-FZD Protein Interactions Reveals Functional Selectivity by Distinct WNT-FZD Pairs. J Biol Chem.

[69] Cheung LYM, Camper SA (2020). PROP1-dependent retinoic acid signaling regulates developmental pituitary morphogenesis and hormone expression. Endocrinology.

[70] Ruf-Zamojski F, Zhang Z, Zamojski M, Smith GR, Mendelev N, Liu H, Nudelman G, Moriwaki M, Pincas H, Castanon RG, Nair VD, Seenarine N, Amper MAS, Zhou X, Ongaro L, Toufaily C, Schang G, Nery JR, Bartlett A, Aldridge A, Jain N, Childs GV, Troyanskaya OG, Ecker JR, Turgeon JL, Welt CK, Bernard DJ, Sealfon SC (2021). Single nucleus multi-omics regulatory landscape of the murine pituitary. Nat Commun.

[71] Ho CC, Bernard DJ (2009). Bone Morphogenetic Protein 2 Signals via BMPR1A to Regulate Murine Follicle-Stimulating Hormone Beta Subunit Transcription. Biol Reprod.

[72] Zhou X, Wang Y, Ongaro L, Boehm U, Kaartinen V, Mishina Y, Bernard DJ (2016). Normal gonadotropin production and fertility in gonadotrope-specific Bmpr1a knockout mice. J Endocrinol.

[73] Haston S, Pozzi S, Carreno G, Manshaei S, Panousopoulos L, Gonzalez-Meljem JM, Apps JR, Virasami A, Thavaraj S, Gutteridge A, Forshew T, Marais R, Brandner S, Jacques TS, Andoniadou CL, Martinez-Barbera JP (2017). MAPK pathway control of stem cell proliferation and differentiation in the embryonic pituitary provides insights into the pathogenesis of papillary craniopharyngioma. Development.

[74] Yoshida S, Kato T, Higuchi M, Chen M, Ueharu H, Nishimura N, Kato Y (2015). Localization of juxtacrine factor ephrin-B2 in pituitary stem/progenitor cell niches throughout life. Cell Tissue Res.

[75] Higuchi M, Yoshida S, Kanno N, Mitsuishi H, Ueharu H, Chen M, Nishimura N, Kato T, Kato Y (2017). Clump formation in mouse pituitary-derived non-endocrine cell line Tpit/F1 promotes differentiation into growth-hormone-producing cells. Cell Tissue Res.

[76] Jing XF, Miyajima M, Sawada T, Chen QF, Iida K, Furushima K, Arai D, Chihara K, Sakaguchi K (2012). Crosstalk of Humoral and Cell-Cell Contact-Mediated Signals in Postnatal Body Growth. Cell Rep.

[77] Haedo MR, Gerez J, Fuertes M, Giacomini D, Paez-Pereda M, Labeur M, Renner U, Stalla GK, Arzt E (2009). Regulation of Pituitary Function by Cytokines. Horm Res.

[78] Missale C, Nash SR, Robinson SW, Jaber M, Caron MG (1998). Dopamine receptors: From structure to function. Physiol Rev.

[79] Marques P, Grossman AB, Korbonits M (2020). The tumour microenvironment of pituitary neuroendocrine tumours. Front Neuroendocrinol.

[80] Akita S, Readhead C, Stefaneanu L, Fine J, TampanaruSarmesiu A, Kovacs K, Melmed S (1997). Pituitary-directed leukemia inhibitory factor transgene forms Rathke's cleft cysts and impairs adult pituitary function - A model for human pituitary Rathke's cysts. J Clin Investig.

[81] Chesnokova V, Melmed S (2000). Leukemia inhibitory factor mediates the hypothalamic pituitary adrenal axis response to inflammation. Endocrinology.

[82] Kariagina A, Romanenko D, Ren SG, Chesnokova V (2004). Hypothalamic-pituitary cytokine network. Endocrinology.

[83] Ware CB, Kariagina A, Zonis S, Alon D, Chesnokova V (2005). Leukemia inhibitory factor signaling is implicated in embrionic development of the HPA axis. FEBS Lett.

[84] Yano H, Readhead C, Nakashima M, Ren SG, Melmed S (1998). Pituitary-directed leukemia inhibitory factor transgene causes Cushing's syndrome: Neuro-immune-endocrine modulation of pituitary development. Mol Endocrinol.

[85] Lodge EJ, Santambrogio A, Russell JP, Xekouki P, Jacques TS, Johnson RL, Thavaraj S, Bornstein SR, Andoniadou CL (2019). Homeostatic and tumourigenic activity of SOX2+ pituitary stem cells is controlled by the LATS/YAP/TAZ cascade. Elife.

[86] Brinkmeier ML, Bando H, Camarano AC, Fujio S, Yoshimoto K, de Souza FSJ, Camper SA (2020). Rathke's cleft-like cysts arise from Isl1 deletion in murine pituitary progenitors. J Clin Investig.

[87] Missale C, Losa M, Boroni F, Giovanelli M, Balsari A, Spano PF (1995). Nerve growth factor and bromocriptine: a sequential therapy for human bromocriptine-resistant prolactinomas. Br J Cancer.

[88] Missale C, Losa M, Sigala S, Balsari A, Giovanelli M, Spano P (1996). Nerve growth factor controls proliferation and progression of human prolactinoma cell lines through an autocrine mechanism. Mol Endocrinol.

[89] Chabot JG, Walker P, Pelletier G (1986). Distribution of epidermal growth factor binding sites in the adult rat anterior pituitary gland. Peptides.

[90] Fan Z, Baselga J, Masui H, Mendelsohn J (1993). Antitumor Effect of Antiepidermal Growth-Factor Receptor Monoclonal-Antibodies Plus Cis-Diamminedichloroplatinum on Well Established A431 Cell Xenografts. Can Res.

[91] Missale C, Spano PF (1998). Growth factors in the pathogenesis of prolactin-secreting tumors. J Endocrinol Invest.

[92] Roh M, Paterson AJ, Asa SL, Chin E, Kudlow JE (2001). Stage-sensitive blockade of pituitary somatomammotrope development by targeted expression of a dominant negative epidermal growth factor receptor in transgenic mice. Mol Endocrinol.

[93] Cox B, Roose H, Vennekens A, Vankelecom H (2017). Pituitary stem cell regulation: who is pulling the strings?. J Endocrinol.

[94] Kobrin MS, Asa SL, Samsoondar J, Kudlow JE (1987). Alpha-transforming growth factor in the bovine anterior pituitary gland: secretion by dispersed cells and immunohistochemical localization. Endocrinology.

[95] Fan X (1995). Epidermal growth factor and transforming growth factor-alpha messenger ribonucleic acids and their receptors in the rat anterior pituitary: localization and regulation. Endocrinology.

[96] Missale C, Spano P (1998). Nerve growth factor in pituitary development and pituitary tumors. Front Neuroendocrinol.

[97] Timaxian C, Raymond-Letron I, Bouclier C, Gulliver L, Le Corre L, Chebli K, Guillou A, Mollard P, Balabanian K, Lazennec G (2020). The health status alters the pituitary function and reproduction of mice in a Cxcr2-dependent manner. Life Sci Alliance.

[98] Vennekens A, Laporte E, Hermans F, Cox B, Modave E, Janiszewski A, Nys C, Kobayashi H, Malengier-Devlies B, Chappell J, Matthys P, Garcia MI, Pasque V, Lambrechts D, Vankelecom H (2021). Interleukin-6 is an activator of pituitary stem cells upon local damage, a competence quenched in the aging gland. Proc Natl Acad Sci.

[99] Sapochnik M, Fuertes M, Arzt E (2017). Programmed cell senescence: role of IL-6 in the pituitary. J Mol Endocrinol.

[100] Gaston-Massuet C, Andoniadou CL, Signore M, Jayakody SA, Charolidi N, Kyeyune R, Vernay B, Jacques TS, Taketo MM, Le Tissier P, Dattani MT, Martinez-Barbera JP (2011). Increased wingless (Wnt) signaling in pituitary progenitor/stem cells gives rise to pituitary tumors in mice and humans. Proc Natl Acad Sci USA.

[101] Gonzalez-Meljem JM, Haston S, Carreno G, Apps JR, Pozzi S, Stache C, Kaushal G, Virasami A, Panousopoulos L, Mousavy-Gharavy SN, Guerrero A, Rashid M, Jani N, Goding CR, Jacques TS, Adams DJ, Gil J, Andoniadou CL, Martinez-Barbera JP (2017). Stem cell senescence drives age-attenuated induction of pituitary tumours in mouse models of paediatric craniopharyngioma. Nat Commun.

[102] Kotliar D, Veres A, Nagy MA, Tabrizi S, Hodis E, Melton DA, Sabeti PC (2019). Identifying gene expression programs of cell-type identity and cellular activity with single-cell RNA-Seq. Elife.

[103] Mayran A, Sochodolsky K, Khetchoumian K, Harris J, Gauthier Y, Bemmo A, Balsalobre A, Drouin J (2019). Pioneer and nonpioneer factor cooperation drives lineage specific chromatin opening. Nat Commun.

[104] Mayran A, Khetchoumian K, Hariri F, Pastinen T, Gauthier Y, Balsalobre A, Drouin J (2018). Pioneer factor Pax7 deploys a stable enhancer repertoire for specification of cell fate. Nat Genet.

[105] Budry L, Balsalobre A, Gauthier Y, Khetchoumian K, L'Honore A, Vallette S, Brue T, Figarella-Branger D, Meij B, Drouin J (2012). The selector gene Pax7 dictates alternate pituitary cell fates through its pioneer action on chromatin remodeling. Genes Dev.

[106] Caetano AJ, Yianni V, Volponi A, Booth V, D'Agostino EM, Sharpe P (2021). Defining human mesenchymal and epithelial heterogeneity in response to oral inflammatory disease. Elife.

[107] Gulati GS, Sikandar SS, Wesche DJ, Manjunath A, Bharadwaj A, Berger MJ, Ilagan F, Kuo AH, Hsieh RW, Cai S, Zabala M, Scheeren FA, Lobo NA, Qian D, Yu FB, Dirbas FM, Clarke MF, Newman AM (2020). Single-cell transcriptional diversity is a hallmark of developmental potential. Science.

[108] Zhang S, Cui Y, Ma X, Yong J, Yan L, Yang M, Ren J, Tang F, Wen L, Qiao J (2020). Single-cell transcriptomics identifies divergent developmental lineage trajectories during human pituitary development. Nat Commun.

[109] Pott S, Lieb JD (2015). Single-cell ATAC-seq: strength in numbers. Genome Biol.

[110] Cheung LYM, Rizzoti K (2020). Cell population characterization and discovery using single-cell technologies in endocrine systems. J Mol Endocrinol.

[111] Cheung LYM, George AS, McGee SR, Daly AZ, Brinkmeier ML, Ellsworth BS, Camper SA (2018). Single-Cell RNA Sequencing Reveals Novel Markers of Male Pituitary Stem Cells and Hormone-Producing Cell Types. Endocrinology.

[112] Fletcher PA, Smiljanic K, Previde RM, Iben JR, Li TW, Rokic MB, Sherman A, Coon SL, Stojilkovic SS (2019). Cell type- and sex-dependent transcriptome profiles of rat anterior pituitary cells. Front Endocrinol.

[113] Chen QY, Leshkowitz D, Blechman J, Levkowitz G (2020). Single-cell molecular and cellular architecture of the mouse neurohypophysis. Eneuro.

[114] Ho YG, Hu P, Peel MT, Chen SX, Camara PG, Epstein DJ, Wu H, Liebhaber SA (2020). Single-cell transcriptomic analysis of adult mouse pituitary reveals sexual dimorphism and physiologic demand-induced cellular plasticity. Protein Cell.

[115] Lopez JP, Brivio E, Santambrogio A, De Donno C, Kos A, Peters M, Rost N, Czamara D, Brückl TM, Roeh S, Pöhlmann ML, Engelhardt C, Ressle A, Stoffel R, Tontsch A, Villamizar JM, Reincke M, Riester A, Sbiera S, Fassnacht M, Mayberg HS, Craighead WE, Dunlop BW, Nemeroff CB, Schmidt MV, Binder EB, Theis FJ, Beuschlein F, Andoniadou CL, Chen A (2021). Single-cell molecular profiling of all three components of the HPA axis reveals adrenal ABCB1 as a regulator of stress adaptation. Sci Adv.

[116] Moncho-Amor V, Chakravarty P, Galichet C, Matheu A, Lovell-Badge R, Rizzoti K (2021) SOX2 is required independently in both stem and differentiated cells for pituitary tumorigenesis in p27-null mice. Proc Natl Acad Sci USA 118(7) 10.1073/pnas.2017115118 PMC7896314 33574062

[117] Cui Y, Li C, Jiang Z, Zhang S, Li Q, Liu X, Zhou Y, Li R, Wei L, Li L, Zhang Q, Wen L, Tang F, Zhou D (2021). Single-cell transcriptome and genome analyses of pituitary neuroendocrine tumors. Neuro-Oncology.

[118] Allensworth-James M, Banik J, Odle A, Hardy L, Lagasse A, Moreira ARS, Bird J, Thomas CL, Avaritt N, Kharas MG, Lengner CJ, Byrum SD, MacNicol MC, Childs GV, MacNicol AM (2021). Control of the Anterior Pituitary Cell Lineage Regulator POU1F1 by the Stem Cell Determinant Musashi. Endocrinology.

[119] Zhang JN, Lv C, Mo CH, Liu M, Wan YP, Li J, Wang YJ (2021). Single-cell RNA sequencing analysis of chicken anterior pituitary: a bird's-eye view on vertebrate pituitary. Front Physiol.

[120] Siddique K, Ager-Wick E, Fontaine R, Weltzien FA, Henkel CV (2021). Characterization of hormone-producing cell types in the teleost pituitary gland using single-cell RNA-seq. Sci Data.

[121] Zhang Z, Zamojski M, Smith GR, Willis TL, Yianni V, Mendelev N, Pincas H, Seenarine N, Amper MAS, Vasoya M, Cheng WS, Zaslavsky E, Nair VD, Turgeon JL, Bernard DJ, Troyanskaya OG, Andoniadou CL, Sealfon SC, Ruf-Zamojski F (2022). Single nucleus transcriptome and chromatin accessibility of postmortem human pituitaries reveal diverse stem cell regulatory mechanisms. Cell Rep.

[122] Laporte E, Hermans F, De Vriendt S, Vennekens A, Lambrechts D, Nys C, Cox B, Vankelecom H (2022). Decoding the activated stem cell phenotype of the neonatally maturing pituitary. Elife.

[123] Mogi C, Goda H, Mogi K, Takaki A, Yokoyama K, Tomida M, Inoue K (2005). Multistep differentiation of GH-producing cells from their immature cells. J Endocrinol.

[124] Cohen LE, Zanger K, Brue T, Wondisford FE, Radovick S (1999). Defective retinoic acid regulation of the Pit-1 gene enhancer: A novel mechanism of combined pituitary hormone deficiency. Mol Endocrinol.

[125] Palomino T, Barettino D, Aranda A (1998). Role of GHF-1 in the regulation of the rat growth hormone gene promoter by thyroid hormone and retinoic acid receptors. J Biol Chem.

[126] Denef C (2008). Paracrinicity: The story of 30 years of cellular pituitary crosstalk. J Neuroendocrinol.

[127] Efremova M, Vento-Tormo M, Teichmann SA, Vento-Tormo R (2020). Cell PhoneDB: inferring cell-cell communication from combined expression of multi-subunit ligand-receptor complexes. Nat Protoc.

[128] Browaeys R, Saelens W, Saeys Y (2020). NicheNet: modeling intercellular communication by linking ligands to target genes. Nat Methods.

[129] Cabello-Aguilar S, Alame M, Kon-Sun-Tack F, Fau C, Lacroix M, Colinge J (2020). SingleCellSignalR: inference of intercellular networks from single-cell transcriptomics. Nucleic Acids Res.

[130] Jin SQ, Guerrero-Juarez CF, Zhang LH, Chang I, Ramos R, Kuan CH, Myung P, Plikus MV, Nie Q (2021). Inference and analysis of cell-cell communication using CellChat. Nat Commun.

[131] Fletcher PA, Smiljanic K, Previde RM, Constantin S, Sherman AS, Coon SL, Stojilkovic SS (2022) The astroglial and stem cell functions of adult rat folliculostellate cells. Glia 1-24 10.1002/glia.24267 PMC9772113 36093576

